# Adhesion and Friction in Biological and Bioinspired Systems

**DOI:** 10.3390/biomimetics11050295

**Published:** 2026-04-24

**Authors:** Thies H. Büscher, Stanislav N. Gorb

**Affiliations:** Functional Morphology and Biomechanics, Zoological Institute, Kiel University, D-24118 Kiel, Germany; sgorb@zoologie.uni-kiel.de

In nature, biological systems employ adhesion and friction in various contexts. Organisms have evolved a variety of specialized biological attachment systems that enhance adhesion and friction, enabling them to attach to diverse substrates firmly or to interconnect different parts of their own bodies [1]. Such systems are widespread in nature and enable organisms to attach temporarily, facilitating locomotion and mating [2], or permanently aiding in settlement [3] to various substrates. Conversely, in other scenarios, such as within joints or on animal surfaces exposed to aquatic environments, contact forces are minimized through various mechanisms to reduce adhesion and friction, for example, to reduce abrasive wear [4], drag [5], or contamination [6]. The functional principles underlying these systems are based on multiple mechanisms hierarchically integrated across different length scales, and both phenomena are tailored through an interplay of (1) surface chemistry, (2) specific mechanical material properties, (3) fluid or solid secretions, and (4) sophisticated micro- and nanostructures.

Numerous adhesion- and friction-related mechanisms that maximize or minimize contact forces have independently evolved across various taxa, since different organisms must meet unique functional requirements shaped by their specific environmental demands and selective pressures [7]. The link between specialized problem-solving strategies and the application of universal physical principles makes these systems an especially promising subject of study in the field of biomimetics [8]. Examining how these distinct biological systems deal with comparable challenges can help to identify functional constraints, while the wide range of biological strategies offers valuable inspiration for biomimetic design. To date, such phenomena have been investigated in detail only for a limited number of selected biological systems and have received comparatively little attention in biomimetic research.

The aim of this Special Issue is to connect interdisciplinary research on mechanisms of adhesion and friction across biological and bioinspired systems. It seeks to highlight how organisms achieve adhesion enhancement, reduction, or mechanical interlocking through structural, chemical, and mechanical adaptations, and to explore how these principles can inform the design of functional materials and devices. By integrating perspectives from biology, physics, and engineering, the Special Issue aims to deepen our understanding of structure–property relationships and foster innovation in biomimetic applications. The completed Special Issue covers a diverse collection of studies dealing with topics on adhesion and friction, from cell adhesion phenomena via non-specific adhesion to the mechanical interlocking of structures. Additionally, several contributions addressed friction and adhesion reduction. Some contributions provide insight into how surface structure, contact mechanics, and surface chemistry contribute to biological attachment, while others explore bioinspired engineered developments and structure–property relationships in artificial systems.

Together, these contributions showcase research from natural systems and biological insight to applied biomimetic design and engineering realization. The collection underscores how studying the structural and mechanical principles of adhesion, friction, and surface interaction across living organisms can inspire innovative technological solutions. Ranging from biological phenomena, through experimental and simulation-driven understanding, to applied studies and material design concepts, the Special Issue also bridges different disciplines. This line—from biological observation to bioinspired application—reflects the overarching aim of biomimetics: transforming natural mechanisms into functional technologies for diverse industrial and biomedical contexts.

In summary, the reader can expect an overview of recent activities in the field of adhesion- and friction-related phenomena in biomimetics. On the side of property characterization of biological and bioinspired systems, Zhou et al. [9] investigated the role of substrate compliance on the jumping performance in a tree frog species with adhesive toepads. They explored the functional adaptation of tree frog adhesive devices in locomotion and surface interaction between this biological system and its substrates. One step further in the progress of generalization of functional principles is the characterization of properties of functional surfaces that are inspired by a biological template. In this setting, Saccardi et al. [10] investigated the anti-adhesive surfaces they produced based on the mandible microstructures of bees. The mandibles of honeybees are confronted with naturally strong sticking substances (propolis) and avoid persistent adhesion by microstructures and an anti-adhesive coating on the inner side of the mandibles. The authors conducted experiments on a range of artificial replicas of this system to assess the influence of microstructure and surface chemistry on propolis adhesion in this context. Lastly, Ji et al. [11] present a study on bioinspired footwear that is inspired by reptilian adhesive systems. Their development is intended to provide stabilization for foot placement under microgravity in space application contexts and, furthermore, aids in the physiological protection of muscle deterioration in weightless environments.

Two papers present studies on experimental and methodological advances in the field of adhesion- and friction-related biomimetics. Tabraue-Rubio et al. [12] developed and compared methods for binding cells to functionalized AFM probes, advancing nanoscale measurement and biointerface characterization methodologies on the cellular level. Ferreira et al. [13] studied adhesive performance at the macroscale in horseshoe bonding through mechanical and numerical analysis. Combining engineering testing with numerical modeling to understand composite joining, the authors compared different adhesives used in the equine industry to establish methods for reliable and less invasive horseshoeing procedures.

In the field of computational and simulation studies, the paper by Liu et al. [14] offers insight into surface-fluid interactions that minimize energy loss through numerical analyses of drag reduction in biomimetic microstructured surfaces.

Finally, contributions on applied biomimetics focused on advances in concepts in engineering design. Mishnaevsky et al. [15] propose a bio-inspired concept for sustainable wind turbine blades, integrating natural adhesive strategies into large-scale composite design. Among the main challenges of sustainable wind energy technology is the contrasting demand for the durability of wind turbine blades versus the recyclability of their components. By developing a bio-inspired mechanism combining chemical adhesion and mechanically interlocking fibers, the authors propose a concept to improve the operational duration of wind turbine blades while facilitating the separation of the components for recycling. For application in various contexts, Yang et al. [16] introduced phenolic intervention as a method to tune the properties of food-grade kappa-carrageenan hydrogels. They show that different plant polyphenols are capable of modifying the properties of such hydrogels and present a route for targeted property manipulation via tannic acid and pyrogallol for selective increase in adhesive properties, thermal behavior, and premediated disintegration. In their review article, Kumar et al. [17] provide a comprehensive review of bioinspired tribological materials for sliding, erosive, machining, and energy-absorbing applications, highlighting multifunctionality in performance as a strength for bioinspired engineering.

The contributions in this Special Issue from both biological and engineering perspectives offer valuable interdisciplinary connections, reinforcing the strong link between natural principles and their biomimetic applications. They showcase the breadth of current research in biological and bioinspired systems and highlight promising directions for future investigations. We are grateful for the wide range of contributions to this Special Issue and sincerely thank all colleagues who participated in it by writing their contributions and/or by reviewing submitted manuscripts. Their commitment made it possible to assemble this collection of articles addressing biomimetic questions on adhesion and friction from multiple scientific viewpoints.
